# MR guided high intensity focused ultrasound (MRgHIFU) for treating recurrent gynaecological tumours: a pilot feasibility study

**DOI:** 10.1259/bjr.20181037

**Published:** 2019-04-20

**Authors:** Sharon L Giles, Georgios Imseeh, Ian Rivens, Gail R ter Haar, Alexandra Taylor, Nandita M deSouza

**Affiliations:** 1 The CRUK Cancer Imaging Centre, The Institute of Cancer Research and The Royal Marsden Hospital, London, United Kingdom; 2 Department of Gynae-Oncology, The Royal Marsden Hospital, London, United Kingdom; 3 Therapeutic Ultrasound, The Institute of Cancer Research, London, United Kingdom

## Abstract

**Objective:**

To assess the feasibility of targeting recurrent gynaecological tumours with MR guided high intensity focused ultrasound (MRgHIFU).

**Methods:**

20 patients with recurrent gynaecological tumours were prospectively scanned on a Philips/Profound 3 T Achieva MR/ Sonalleve HIFU system. Gross tumour volume (GTV) and planning target volume (PTV) were delineated on *T*_2_W and diffusion-weighted imaging (DWI). Achievable treatment volumes that (i) assumed bowel and/or urogenital tract preparation could be used to reduce risk of damage to organs-at-risk (TV_optimal_), or (ii) assumed no preparations were possible (TV_no-prep_) were compared with PTV on virtual treatment plans. Patients were considered treatable if TV_optimal_ ≥ 50 % PTV.

**Results:**

11/20 patients (55%) were treatable if preparation strategies were used: nine had central pelvic recurrences, two had tumours in metastatic locations. Treatable volume ranged from 3.4 to 90.3 ml, representing 70 ± 17 % of PTVs. Without preparation, 6/20 (30%) patients were treatable (four central recurrences, two metastatic lesions). Limiting factors were disease beyond reach of the HIFU transducer, and bone obstructing tumour access. DWI assisted tumour outlining, but differences from *T*_2_W imaging in GTV size (16.9 ± 23.0%) and PTV location (3.8 ± 2.8 mm in phase-encode direction) limited its use for treatment planning.

**Conclusions:**

Despite variation in size and location within the pelvis, ≥ 50 % of tumour volumes were considered targetable in 55 % patients while avoiding adjacent critical structures. A prospective treatment study will assess safety and symptom relief in a second patient cohort.

**Advances in knowledge:**

Target size, location and access make MRgHIFU a viable treatment modality for treating symptomatic recurrent gynaecological tumours within the pelvis.

## Introduction

Patients with recurrent gynaecological malignancies frequently have symptomatic localised pelvic disease and high morbidity and mortality.^1–3^ If uncontrolled, symptoms (*e.g.* pain, vaginal bleeding and/or discharge, bladder and/or bowel symptoms, fistulae, lymphoedema^4^) are progressive, and adversely impact quality of life, particularly in those previously treated with external beam radiotherapy (EBRT) or brachytherapy.^5^ Treatment options are limited by the site and extent of recurrence, and the nature of previous treatment. Radical radiotherapy, usually administered for recurrence following surgery, carries a risk of treatment-related toxicities, and often cannot be used after previous radiotherapy.^6^ In isolated relapse, pelvic exenteration may potentially be curative, but carries high (40–80%) morbidity, with a 20–40% 5-year survival.^7,8^ Used palliatively, radiotherapy and surgery may achieve symptomatic control, but induce acute bowel and bladder problems, while palliative chemotherapy is <20% effective at symptomatic relief within a previously irradiated field.^9^

High intensity focused ultrasound (HIFU) has been used for many years to treat benign gynaecological disease.^10^ The thermally ablative mechanism of action is well understood,^11^ and as HIFU does not use ionising radiation, it can be delivered to a previously irradiated field without risking additional radiation toxicity. Therefore, HIFU offers a potential alternative treatment in patients with recurrent gynaecological tumours, particularly as recurrence often occurs at a single site.^12^ However, evidence for efficacy of HIFU treatment of these tumours is extremely limited, comprising only two case-reports in two females with recurrent cervical tumours.^13,14^ In both cases, patients gained significant short-term improvements in pain and/or bleeding without adverse events, but long-term follow up was unavailable. Whilst these case reports are encouraging, and there is extensive evidence for HIFU treatment of uterine fibroids, the anatomical location of recurrent tumours is variable, and pelvic anatomy may be distorted by prior treatments. Consequently, the feasibility of treating these lesions with a conventional extracorporeal HIFU system needs to be established. The purpose of this pilot study, therefore, was to assess the feasibility of targeting recurrent gynaecological tumours with MRgHIFU, prior to undertaking a treatment study. We set an ablation target of ≥50% of the lesion volume as a criterion for treatability.

## Methods

### Study design

This was the feasibility arm of a prospective single-centre study investigating MRgHIFU for treatment of recurrent gynaecological malignancies (NCT02714621).^15^ Patients were positioned on a Sonalleve HIFU clinical system (Profound Medical Corp, Ontario, Canada), and scanned within the bore of a 3T Achieva MR (Philips Healthcare, Best, The Netherlands). Images enabled virtual treatment plans to be constructed using Sonalleve treatment planning software. An arbitrary threshold of targeting ≥50% of a target lesion volume without damage to surrounding organs at risk (OARs) in ≥20% of the patients was set as the criterion for success to proceed to the treatment arm of NCT02714621.

### Study Population

Over a 20-month period from June 2016 to February 2018, 20 adult females with a proven diagnosis of locally recurrent gynaecological malignancy were recruited. Eligibility criteria for the study are provided in Table 1. Medical history, symptoms and any potential risk factors for anaesthesia were documented. Each patient was given a study-specific patient information sheet, before providing written, informed consent to participate.

**Table 1.** Eligibility criteria for the study. Patients’ suitability for other treatments was not a consideration in this planning study assessing treatment feasibility

**Inclusion criteria**	**Exclusion criteria**
Adult patients (≥18 years) with confirmed recurrent pelvic gynaecological malignancy	MRI contra indicated (*e.g.* by incompatible metal implants, claustrophobia or BMI precludes accommodation in the MRI scanner)
Intended target visible on non-contrast MRI	Pregnancy
Intended target potentially within transducer range (1–10 cm beneath the skin)	Patient lacking capacity to consent for study
Patient willing to undergo study-specific MRI, not necessary for clinical care	
Patients willing to provide details of prior medical and treatment history	

### Patient preparation for MRI

Patients were encouraged to drink water, and refrain from micturition prior to scanning, if a partly full bladder was thought likely to be helpful for targeting their region of disease. This was not pursued if the patient had concerns about incontinence or discomfort; instead review of the medical notes determined whether intervention (*e.g.* bladder filling by urinary catheter) would have been possible. No patients underwent preparation to vagina or bowel; instead we determined whether the region close to the target disease was likely to be amenable to clinically acceptable preparation strategies (*e.g.* aspiration of air, or filling with ultrasound gel).

### Patient positioning and MR image acquisition

Patients were placed in a potential treatment position that minimised the distance between the target lesion and the centre of the acoustic window built into the treatment couch. Images were acquired with the standard Sonalleve HIFU window and pelvis coils. *T*_2_- and *T*_1_-weighted (*T*_2_W, *T*_1_W) sequences were acquired axially through the whole pelvis, and used to plan high-resolution 3D *T*_2_W imaging. This provided orthogonal image reconstructions relative to any planned position and angle of the transducer at the target. As tumour margins can be difficult to distinguish on *T*_1_W or *T*_2_W imaging on a background of post-surgical or radiotherapy-induced tissue changes, diffusion-weighted imaging (DWI) was also acquired, because it provides better tumour-to-normal-tissue contrast.^16^ Summary sequence parameters are provided in Table 2. If disease could potentially have been targeted from different approaches, patients underwent repeat scanning of the *T*_2_W imaging only in the additional treatment positions.

**Table 2.** *T*_*2*_W and diffusion-weighted imaging (DWI) sequence parameters. DWI sequences were voxel matched, slice matched to the 2D *T*_*2*_W imaging, and corresponded with every third slice of the higher resolution 3D *T*_*2*_W imaging

**Parameter**	**2D TSE T2W**	**3D TSE T2W**	**EPI-DWI**
TR (ms)	3620	1500	9000
TE (ms)	90	165	65
FA (^o^)	90	90	90
Fat suppression	-	-	SPIR & SSGR
EPI/TSE factor	16	67	69
SENSE factor	1.5 (RL)	1.5 (RL), 2.0 (FH)	1.6 (RL)
b values (s/mm^2^)	-	-	0, 100, 700
rBW (Hz/pix)	-		26.3
Shim	Auto	Volume	Pencil beam volume
Voxel size (mm^3^)	1.0 × 1.0×4.5	1.5 × 1.5×1.5	3.5 × 3.5×4.5
FOV (mm)	280 × 300×180	250 × 250×200	300 × 327×185
NSA	1	1	3
Number slices	40	133	41
Slice orientation	transverse	transverse	transverse
Scan duration min:sec	02:32	02:13	4:48

### Measurement of gross tumour volume (GTV)

The maximum dimensions of the tumour, and its minimum and maximum depths from the skin were measured on 2D *T*_*2*_W imaging. GTV was calculated by manually drawing a region of interest (ROI) conformal to the tumour on every slice demonstrating it, using in-house software (Adept, The Institute of Cancer Research ), and summing all ROI areas multiplied by the slice thickness and slice gap.

### Defining the planning target volume (PTV)

**Figure 1.**
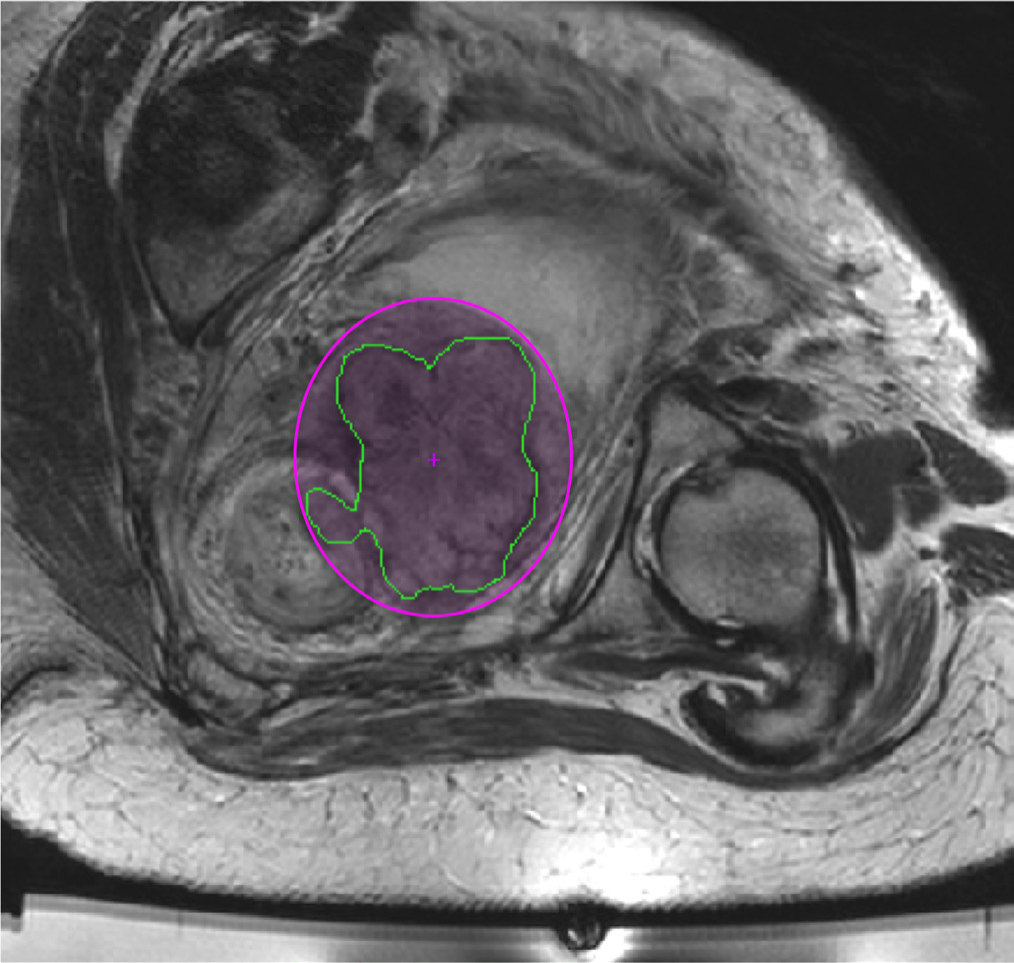
The planning target volume (PTV) (pink ellipse) closely encompassed the gross tumour volume (GTV) (green outline), shown here on a single slice of the 2D *T*_*2*_W imaging. The co-ordinates (AP, LR and FH) of the PTV centre (pink cross) were read using a pixel information tool on each of 3 orthogonal imaging planes reconstructed from the 3D *T*_*2*_W imaging. Available Sonalleve treatment planning software did not allow the slice-by slice GTV outlines to be displayed within PTVs.

An ellipsoidal PTV (the only option in the available software) that closely encompassed the GTV (Figure 1) was defined using the Sonalleve treatment planning software on orthogonal multi planar reformats of the 3D *T*_2_W imaging, with reference to all available imaging. Its volume was provided by the treatment planning software. In 16/20 patients, a single PTV was used to encompass the GTV. In the remaining four, better conformity to eccentrically shaped tumour regions was obtained by using two partially overlapping PTVs, whose non-overlapped sum represented the final PTV.

### Assessing feasibility of treatment

**Figure 2.**
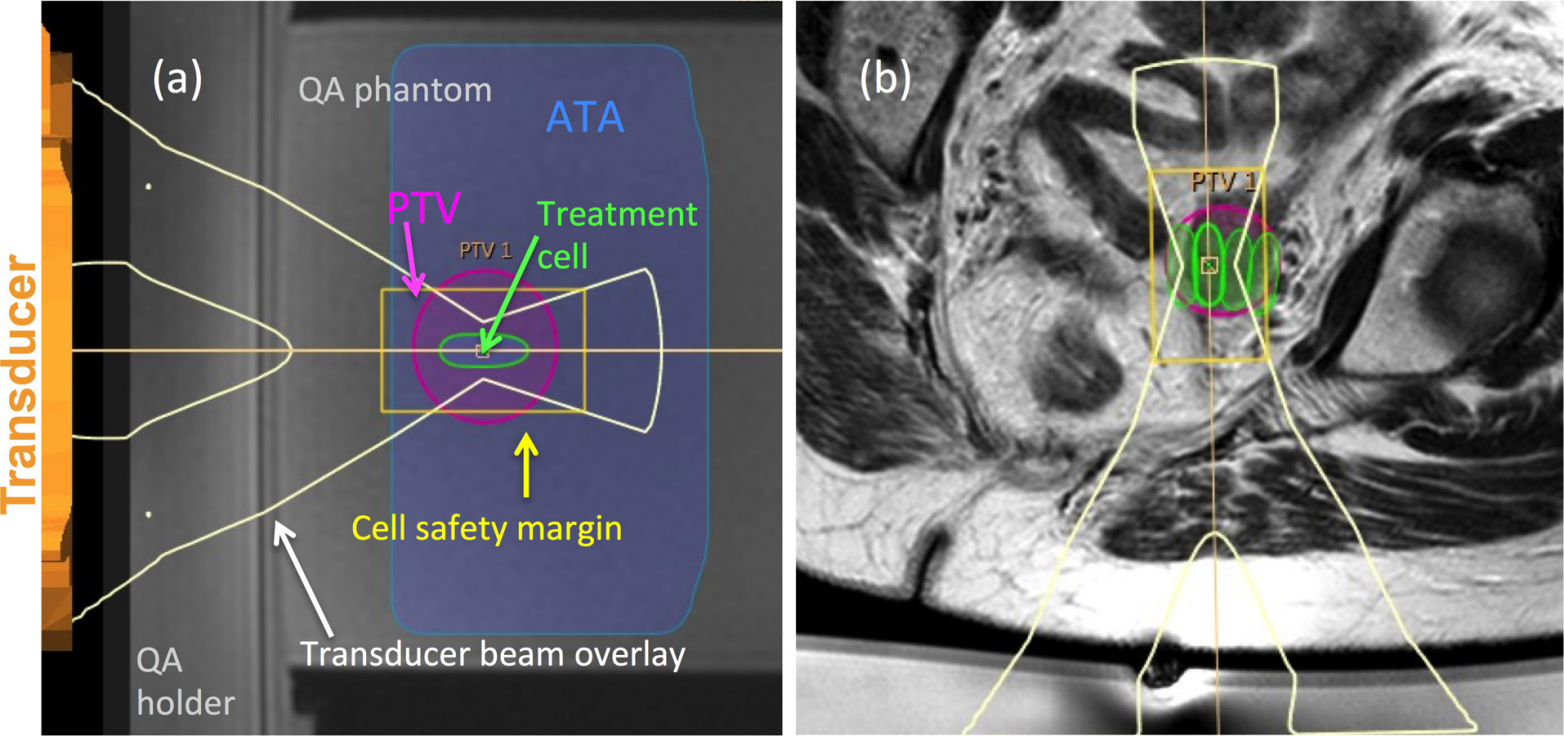
Sonalleve treatment planning tools are shown overlaid on (a) an image of the QA phantom (for clarity), and (b) a patient’s *T*_*2*_W image. Treatment cells (green ellipses) are positioned within the planning target volume (PTV) (pink ellipse). The transducer beam overlay is indicated by the white y-shaped graphics, with the region at greatest risk of heating (cell safety margin) indicated by the yellow rectangle. The available target area (ATA) indicates the region within range of the transducer. Each cell was safety checked to determine whether bone or heat sensitive non-target regions lay within the transducer beam overlay, or within the cell safety margin.

Appropriately sized HIFU treatment cells (4, 8, 12, 14, 16 mm diameter; 0.1, 0.8, 2.5, 3.8, 5.6 ml volume)^17^ were prescribed manually, to cover the entire PTV. This was first done without considering acoustic obstacles in the beam path or OARs, but by simply ensuring that cells were within focal range of the transducer (Figure 2). These cells were then safety-checked to determine if they could be delivered without obstruction or risk, in particular whether: (i) bone was in the beam path (denoted by the beam overlay shown in Figure 2), (ii) MR-visible nerves were within the cell safety margin (Figure 2), or (iii) there was high risk of damage to the bowel or urogenital tract. This third assessment was made under the assumption that preparation could be used to minimise the risk of damage if these organs were located in, or close to the beam path, but not if they were within the cell safety margin. Total volume of these safety-checked cells (TV_optimal_), the number and size of HIFU treatment cells, and estimated treatment time was recorded. A second treatment plan (TV_no-prep_) derived from TV_optimal_, assumed that no interventional preparations were possible.

### Comparing *T*_2_W imaging and DWI for defining GTV and PTV

To estimate any difference in GTV, if measured on DWI, rather than *T*_*2*_W imaging, GTVs were also outlined on DWI (DWI-GTV), done without reference to the *T*_*2*_W images, and using both the *b* = 700 s/mm^2^ images and apparent diffusion coefficient (ADC) maps.

To estimate any difference in PTV volume and location if defined against different imaging techniques, PTVs were also defined separately on *T*_*2*_W imaging (T2-PTV) and DWI (DWI-PTV), and their volumes compared. The central co-ordinates (AP, LR and FH) of each PTV were read using a pixel information tool, and summarised as the mean value of each of the three co-ordinates. Discrepancies between PTV positions were calculated as the magnitude difference values between each set of mean AP, LR and FH co-ordinates.

### Statistical analysis

Statistical analysis was performed using GraphPad Prism software (Version 7, San Diego, CA). D'Agostino & Pearson tests for normality were used to determine whether parametric or non-parametric tests should be used. *p*-values < 0.05 were considered significant.

Log-transformation of GTVs, PTVs and TVs was required to normalise these data, and allow the use of parametric tests. GTVs and PTVs were compared using paired samples t-tests. The difference between PTV and the non-safety-checked treatment volume represented the proportion of disease beyond range of the transducer. TV_optimal_ and TV_no-prep_ were each expressed as percentages of PTV. Patients were considered treatable if TV_optimal_ was ≥50% PTV. Differences in GTV, PTV and the 2 varieties of TV (optimal and no-prep) between treatable and non-treatable patients were compared using unpaired t-tests. Volumes of DWI-GTVs and DWI-PTVs were compared with those outlined on *T*_*2*_W imaging using paired samples t-tests.

## Results

### Patients

Primary tumour types, locations of recurrence, and details of prior treatment are summarised in Table 3. All patients tolerated the required imaging position; in 16 this was supine oblique, one was prone oblique, and one was prone. In the remaining two, more than one position was used. No patient had contraindications to gadolinium administration, but one had potential risk factors for anaesthesia. One patient with disease in an inguinal node who had undergone a prior wide local excision had scar tissue in the HIFU beam path, which would have increased the risk of skin burn if she had gone on to have treatment.

**Table 3.** Patient demographics, prior treatment history, and proposed treatment for recurrent disease. Note that some patients underwent more than one surgical procedure prior to the present recurrence. Only two patients who had radiotherapy for recurrence had also received radiotherapy to the primary tumour

Patient characteristics	
Age (years)	
Mean ± SD	61.4 ± 11.3
Median (range)	61.5 (44–83)
Primary Tumour Site: n (%)	
Cervix	6 (30)
Ovary	5 (25)
Endometrium	5 (25)
Vulva	2 (10)
Primary peritoneal	1 (5)
Bartholin’s Gland	1 (5)
Location of recurrence: n (%)	
Vagina or vault	9 (45)
Pelvic sidewall	4 (20)
Adnexa	2 (10)
Uterus	2 (10)
Inguinal region	1 (5)
Perineal region	1 (5)
Morrison’s Pouch	1 (5)
Interval primary diagnosis to present recurrence (years)	
Median (range)	3.3 (0.8–28.9)
first recurrence / second recurrence / fourth recurrence	*n* = 15/ *n* = 4/ *n* = 1
Prior surgery to primary tumour: n (%)	
Any surgery	18 (90)
Hysterectomy	13 (65)
Local excision	7 (35)
Bilateral Salpingo-oopherectomy	10 (50)
Lymph node dissection	6 (30)
Exenteration	1 (5)
Prior therapy for gynae malignancy: n (%)	
EBRT and brachytherapy to primary tumour	4 (20)
EBRT or brachytherapy to primary tumour	4 (20)
Radiotherapy to prior/present recurrence	8 (40)
Chemotherapy to primary or prior recurrence	14 (70)
Proposed treatment for present recurrence: n (%)	
Radical surgery	4 (20)
Palliative surgery	2 (10)
Radiotherapy / Chemo-radiotherapy	5 (25)
Chemotherapy / Phase 1	5 (25)
Watch and wait or palliative care	4 (20)

### GTV and PTV

Mean ± SD maximum tumour dimensions on *T*_2_W imaging were: 43.5 ± 22.4 mm (AP), 44.2 ± 22.2 mm (LR) and 45.0 ± 23.0 mm (FH). Minimum depth of tumours from the skin was 72.3 ± 21.5 mm; maximum depth was 115.9 ± 27.7 mm. GTV varied across the cohort (mean ± SD: 64.2 ± 82.3 ml, range: 1.1–308.2 ml). PTV was consequently also highly variable (mean ± SD: 83.1 ± 104 ml, range: 1.4–357.8 ml), but was always larger than GTV (difference: 23.8±13.7%).

### Feasibility of treatment

**Figure 3.**
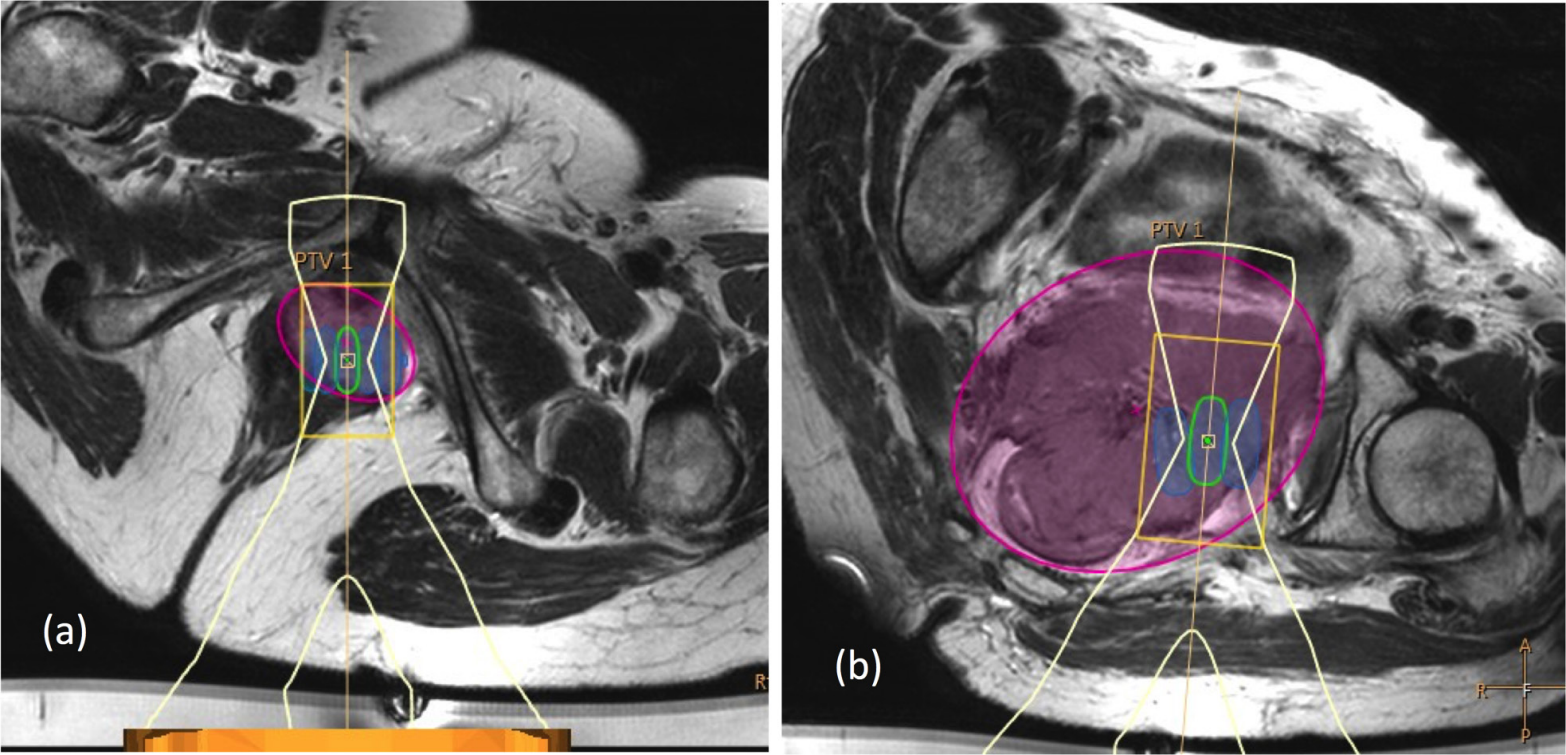
(a) A 64-year-old endometrial cancer patient with a lower vaginal recurrence, who was classified as feasible to treat (GTV = 20.6 ml, PTV = 25.6 ml, TV_optimal_ = 18.4 ml, or 72% of PTV). Treatment was planned using 22 × 8 mm diameter cells, with an estimated treatment time of 50 min. (b) A 65-year-old ovarian cancer patient with a large tumour (GTV = 249 ml, PTV = 359 ml, TV_optimal_ = 122 ml, or 34% PTV). Treatment was not feasible despite using three different approaches (left supine oblique approach only shown).

11/20 patients (55%) were considered treatable if preparation strategies were used: 9 of these had central recurrences and two had tumours in metastatic locations (inguinal nodes and Morrison’s pouch). The potential treatment volume in these patients was 21.9 ± 25.1 ml (range: 3.4–90.3 ml), which represented 70±17% of PTVs. Estimated treatment time was 41.4 ± 29.2 min (not allowing for patient positioning or imaging). Of nine patients not considered treatable with preparation, six also had central recurrences, whilst the remaining three had lesions at the pelvic sidewall. Examples of accessible and non-accessible central recurrences are shown in Figure 3. Potential treatment volumes in the nine non-treatable patients were larger (41.2 ± 46.0 ml, range 0–122 ml) than in the 11 treatable patients, but represented a smaller proportion of PTVs (difference: 23.4±16.8%). This was because both GTV and PTV were significantly smaller in the 11 treatable patients (GTV: 27.2 ± 36.5 ml, range 1.1–128.5 ml; PTV: 34.2 ± 44.5 ml, range 1.4–160.3 ml), compared to the nine who were not treatable (GTV: 109.4 ± 101.1 ml, range 11.2–308.2 ml; PTV: 143.2 ± 125.7 ml, range 12.4–357.8 ml, GTV *p* = 0.006, PTV *p* = 0.005).

Without preparation, 6/20 patients (30%) were still considered treatable: four with central recurrences and two with metastatic lesions. The potential treatable volume in these patients was 33.1 ± 30.2 ml (range: 3.4–90.3 ml), representing 78±20% of PTVs.

### Factors restricting treatment

Even without safety checking, treatment volume was <100% of PTVs in 16/20 patients; the proportion of the PTV beyond reach was 17±25%. In patients assumed to have preparation before treatment, the proportion of PTV that could not be targeted due to (i) bone ranged from 0–66%, (ii) major nerves ranged from 0–51%, and (iii) bladder or bowel ranged from 0–14%. Without preparation, up to 75% of PTVs were not targetable due to risk to bowel, and up to 15% due to risk to bladder. Other restrictions on targeting that could not be mitigated by preparation strategies were (i) close proximity of the femoral artery (3% of PTV for an inguinal lesion), and (ii) the liver and kidney too close to the superior and inferior tumour margins (15% of PTV in Morrison’s pouch).

### Comparing *T*_2_W imaging and DWI for defining GTV and PTV

**Figure 4.**
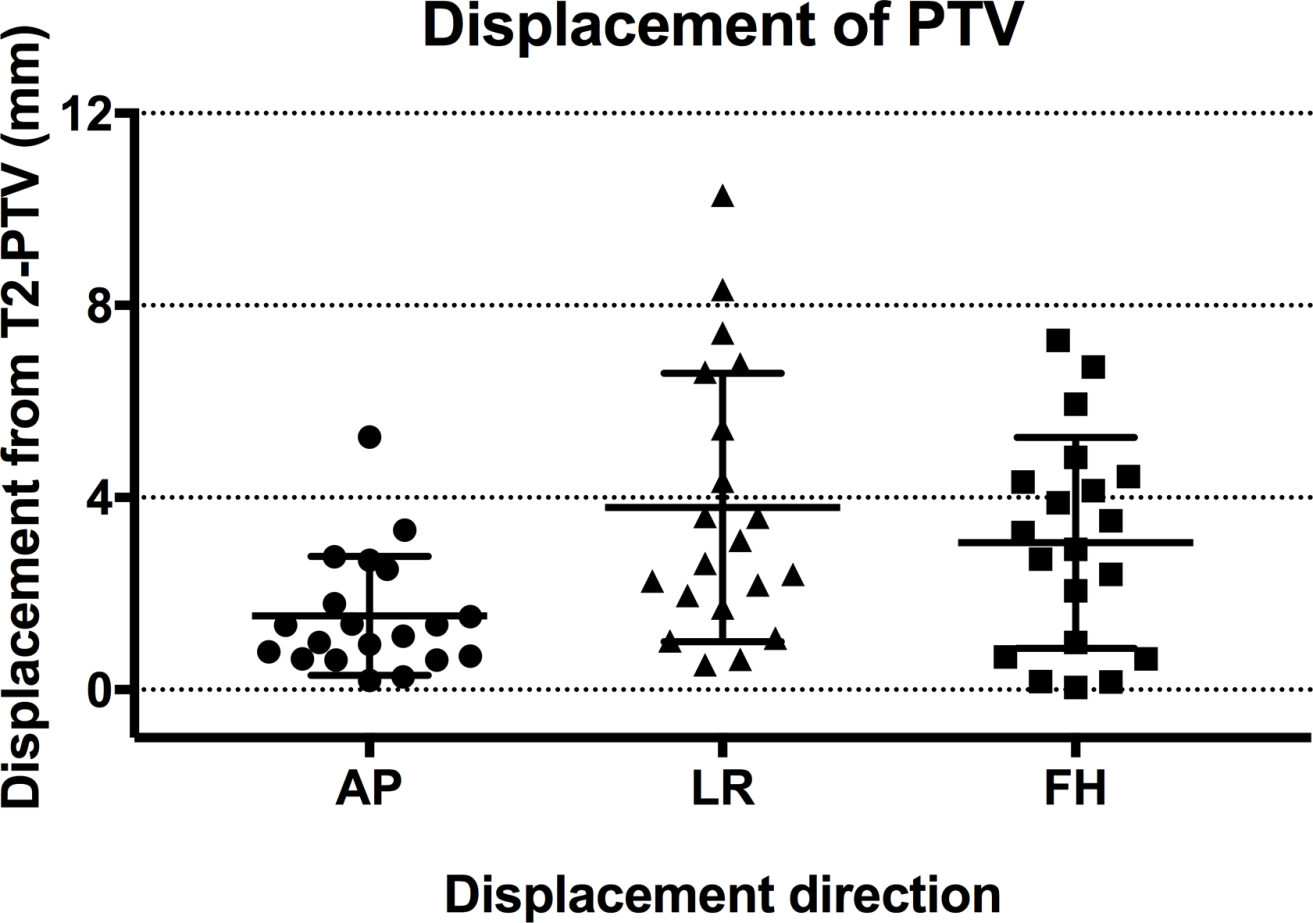
Displacement of DWI-PTVs relative to T2-PTVs. Displacements were worse in the phase-encode (LR) direction, and in the slice-select (FH) direction, than in the frequency-encode (AP) direction.

Tumours were restricted in diffusion relative to surrounding normal tissues in 13/20 patients, which increased their conspicuity on DWI compared to *T*_2_W imaging. DWI-GTV (58.8 ± 87.0 ml, range: 1.0–338.4 ml) was smaller than GTV measured on *T*_*2*_W imaging (difference: 16.9±23.0%, *p* = 0.005). T2-PTVs (82.6 ± 103.2 ml, range: 1.1–355.4 ml) were not significantly different to DWI-PTVs (85.6 ± 118.0 ml, range: 1.6–404.6 ml, *p* = 0.92), or to the PTV defined with reference to all available imaging (*p* = 0.60). However, discrepancies between T2-PTV and DWI-PTV locations were noted (Figure 4). These were worst for the LR co-ordinate (phase-encode imaging direction) (maximum/mean ± SD: 10.3/3.8 ± 2.8 mm). Discrepancy of the FH co-ordinate (slice-encode direction) was 7.3/3.1 ± 2.2 mm, and of the AP co-ordinate (frequency-encode direction) was 5.3/1.5 ± 1.2 mm.

## Discussion

These preliminary data indicate that ≥50% of recurrent gynaecological tumour volumes were targetable in 30% of patients without preparation strategies, or in 55% of patients when strategies for bladder and/or bowel emptying and filling, as used for fibroid treatments were assumed to ensure treatment safety.^18^ We did not actually implement these preparations strategies, but they are already used to promote safety during some radiotherapy regimes to prostate and gynaecological tumours by reducing movement and inter fraction inconsistencies.^19^

The main restrictions for targeting lesions were disease beyond focal range of the transducer, and bone in the beam path (sacrum/coccyx medially and/or superiorly, and iliac bones laterally and/or superiorly). In five patients, bone was a barrier because some of the lesion was too close to a bone surface; in 10, bone could have been avoided by beam angulation, but this placed disease beyond reach of the transducer. The Sonalleve transducer has a focal length of 14 cm, which allows targeting of lesions up to 10 cm below the skin with a vertical beam, or less with an angled beam. To minimize the requirement for beam angulation and maximise the acoustic window through the sciatic notch, most patients were in the supine-oblique position. Ideal angulation was limited by the constraints of the MR scanner bore in some cases, a factor reported by others in a rectal cancer feasibility study.^20^ It would be feasible to treat more patients if a longer focal length transducer were available that did not have a beam profile too wide to be limited by neighbouring bone.

In keeping with recognised patterns of recurrence, the most common target lesion site was the vaginal vault. The three non-accessible vaginal vault lesions were in females with a high body mass index. Recurrent lesions located low in the pelvis, or superficially in inguinal or peritoneal regions, were more accessible. The most challenging locations to target were those higher up the pelvic sidewall, where the bony pelvis obstructed acoustic access, and the proximity of major nerve roots also presented a risk. Lesions located above the level of the sciatic notch in the posterior pelvis are unlikely to be suitable for HIFU treatment using an extra corporeal technique.

As expected, smaller lesions would be more feasible to treat than larger ones. Notwithstanding obstruction by bone, and partial volumes beyond the focal range of the transducer, the potentially large number of sonications, and long treatment times would also make safe treatments of large tumours logistically difficult. In treatments of large fibroids, treatment of just one or two planes within the capsule of the fibroid suffices to achieve the desired >80% non-perfused volume necessary for symptomatic relief.^10^ The same may apply in these malignant lesions where targeting the well-oxygenated periphery may achieve the desired symptomatic relief.

GTVs in this study were defined as for EBRT, representing macroscopic tumour. As with our data, downsizing of the GTV on DWI has been reported in studies prior to EBRT in rectal and anal tumours.^21,22^ DWI may also increase delineation confidence for inexperienced readers, with greater inter observer agreement.^21^ The PTVs in this study differed from those used in planning EBRT, as they included the GTV, but not structures with clinically suspicious involvement (the clinical target volume), or additional margins to account for set-up errors. Our PTVs were always larger than GTVs, but the degree of difference was inconsistent between patients because it was determined by the eccentricity of the GTV. Difference in definition of PTVs for treatment planning of MRgHIFU and EBRT are crucially important to understand during multi disciplinary discussions.

The sequence-dependent differences in GTV in this study did not affect PTV size, but resulted in PTV displacements. These displacements would have resulted in inaccurate cell placement if PTV definition were based on DWI alone. A spatial discrepancy of ±4 mm equates to 2 incorrectly positioned 4 mm diameter treatment cells in every line of that imaging plane (one under treated, one over treated). Our measurements show that this clinically relevant degree of error occurred in the phase-encode direction in approximately half of the patients using DWI. Other studies that have looked at the accuracy of DWI for radiotherapy treatment planning have shown similar results,^22,23^ indicating that DWI assists tumour outlining, but that *T*_2_W imaging is needed for planning accuracy.

A limitation of the PTV analyses was that a single observer defined all PTVs, and that only their volumes and central locations were considered. Available software did not allow comparison of PTV shapes, which could be done using radiotherapy treatment planning software.^22^ However, a fundamental difference is the need to adjust MRgHIFU treatments in real time (to take into account heating patterns seen intra procedurally), rather than following a pre-determined treatment plan. Treatment volumes were measured as the sum of individual treatment cell volumes, and did not consider any overlap between them. However, we mitigated this potential over estimate of treatment volume by using the PTV for the ±50% comparison. Comparison with the smaller GTV would have resulted in one more patient being classified as treatable if optimally prepared, and two more as treatable without preparation. Clinically, the spacing of neighbouring cells would be adjusted to take account of thermal dose dimensions, but this real-time variation could not be assessed here.

This virtual planning study also could not establish the acoustic exposure conditions necessary to achieve ablation. These may be affected by tumour vascularity, but administration of Gadolinium contrast agent to help in estimating this was not justified here. Attenuation or aberration of the ultrasound beam by fat may be another factor in effective dose delivery.^24,25^ It remains to be established whether sufficient energy can be focused at the required depth through fat layers in the pelvis, without excessive risk to surrounding OARs.

## Conclusion

We have showed the potential accessibility of different sites of recurrent gynaecological disease to MRgHIFU, finding that ≥50% of tumours could be targeted in more than half of the patients if clinically acceptable strategies for bowel and urogenital tract emptying and filling were considered. Disease beyond the reach of the transducer, and bone preventing an adequate acoustic window were limitations. The real-time adjustments based on heating patterns seen on intra procedural thermometry, however, will fundamentally determine safety and efficacy of MRgHIFU in this setting, and remain to be established in a treatment study.
